# Research on the learning experience of virtual simulation class experimental teaching and learning based on the perspective of nursing students

**DOI:** 10.1186/s12912-023-01534-z

**Published:** 2023-10-06

**Authors:** Yazhuo Gao, Xuehua Zhu

**Affiliations:** School of Nursing, Zhejiang Chinese Medical University, Hangzhou, 310053 China

**Keywords:** Nursing students, Virtual simulation class, Learning experience, Qualitative research

## Abstract

**Background:**

The enrichment of information technology has impacted traditional teaching modes. The emergence of virtual simulation class experimental teaching software has effectively improved the quality of nursing experiment teaching. The learning experience of virtual simulation class experiment teaching and learning based on the perspective of nursing students is explored to provide a basis for improving related learning effects in the future.

**Methods:**

Fourteen undergraduate nursing students were selected using the purposive sampling method for semi-structured interviews. The Colaizzi seven-step analysis method was used to collate and analyse the interview data.

**Results:**

Two themes and six sub-themes were considered during the data analysis. The two themes were positive experiences and negative experiences. In the positive learning experience, undergraduate nursing students showed a deep memory of authentic and diverse scenes, which presented knowledge in a clearly logical, visualised and stereoscopic manner. Negative experiences are manifested as significantly different learning efficiencies in different grades and subjects, and timing the delivery of teaching feedback is difficult.

**Conclusion:**

Virtual simulation experimental teaching can promote the subjective initiative of nursing students’ learning and promote better coordination and unity in their image and theoretical thinking. Some advantages can be augmented by following the national educational policy, strengthening the information construction, combining the construction of virtual simulation experiments with the discipline’s characteristics and optimising the resources. This paper provides a reference for the future exploration of nursing education and further improving the construction of virtual simulation experimental teaching tools and resources.

Cultivating excellent undergraduate nursing students is inseparable from high-quality experimental teaching. The traditional teaching mode of chalk and blackboard urgently needs reform and innovation due to emerging and novel information technologies and methods. Currently, virtual simulation experimental teaching is widely explored and implemented in various colleges and universities and has received widespread attention.

Many experimental teaching achievements have emerged, the interaction between teachers and students has been significantly enhanced, and the teaching outcomes have rapidly improved. There are many forms of virtual simulation experiments in nursing education, but the network-based virtual simulation experiment is the most widely used form [1].

Based on the randomised, controlled, longitudinal, multisite design of Hayden and other scholars, six-hundred and sixty-six American nursing undergraduates were investigated to consider a control group that received traditional clinical experiment teaching, compared with an intervention group receiving 25% or 50% of the clinical time in combination with virtual simulation experimental instruction.

The results showed that high-quality virtual simulation experiments, instead of approximately half of the traditional clinical instruction, could produce the same teaching outcomes (knowledge, skills, critical thinking) [2]. Therefore, the current nursing regulatory agency recommends using simulation experiments to replace up to 50% of the clinical time [3].

The virtual simulation technology characteristics correspond to the framework of the Jeffries Simulation Theory [4], where simulation means a shift from a teaching focus to a learning focus, in which situation, background, design, educational practice, simulation experience, and results are the core concepts of the whole theory for clinical practice in real life [5].

At present, there is no clear definition of virtual simulation. According to the Healthcare Simulation Dictionary (2020) and the regulations issued by the Ministry of Education of China, the fundamental characteristics of virtual simulation experimental teaching include three aspects: information technology characteristics, highly simulated experimental environment and objects, and meeting undergraduate teaching requirements [6]. Therefore, virtual simulation experimental teaching is based on a highly visual computer application, which combines the latest information technology with education to create a platform that can provide a certain degree of the real world.

As a medium that can convey a strong sense of experience, students can practice individual operations online and evaluate their operating results in real-time. Teachers can access the system through their logins to grasp students’ learning progress, operating time and results, find out the problems students encounter during each scenario as they solve issues to strengthen the positive feedback between students and teachers and help teachers adjust teaching methods in time [7].

Many experimental and model teaching aids that could not be utilised due to space and financial limitations have become possible with the development of virtual simulation experimental teaching software. The subjective initiative of nursing students has also been strengthened. The phenomenon of complete separation of theoretical teaching and experimental teaching has been dramatically alleviated [8–10].

A master plan for the experimental teaching project was produced in 2017 by the Ministry of Education of China. By 2020, approximately 1,000 virtual simulation experimental teaching projects have been built to develop virtual simulation experiments, which simulated and created an experimental simulation environment integrating sight, hearing and touch, broadening the potential of experimental teaching [11].

Such software systems significantly improved nursing students’ clinical system thinking and innovation ability [12, 13], and enhanced the nursing students’ ability for self-assessment, improvement and management of self-learning process. It can be seen that the practical implementation of the project has dramatically promoted the emancipation of the teaching mode of various universities [14, 15].

Some studies have shown the advantages of virtual simulation experiments in skill teaching, such as respiratory diseases related to the removal of ineffective airways and hypoxia, intravenous puncture, medication administration, general assessment skills and other skillsets, augmenting the learning experience of nursing students while improving the knowledge system [16–18].

However, most experiments only evaluate nursing students’ learning outcomes but ignore their subjective feelings. Through semi-structured interviews with nursing undergraduates, this study fully presents the learning experience of virtual simulation class experimental teaching to understand further the necessity and effectiveness of the experimental teaching mode. Such analyses will provide valuable data support for promoting and applying experimental teaching modes.

## Material and methods

### Research methods

This qualitative study was conducted at a university in Zhejiang between June 2022 to July 2022. This study used a purposive sampling method to select 14 nursing undergraduates from the university’s nursing school, including senior undergraduates of 2018, junior undergraduates of 2019 who were about to enter the internship stage and 2020 sophomores.

The inclusion criteria of interviewees were: (1) Voluntary participation in the interview and a signed informed consent form; (2) Good language expression ability; (3) Full-time nursing undergraduates; (4) Students who have participated in the virtual simulation experimental teaching mode.

The number of participants was determined after information saturation was reached during the interview collection when no new topics arose and duplicate responses emerged. The Twelfth interview data inferred has saturation, with two further interviews confirming this outcome. Such a fundamental research principle follows previous qualitative research studies [19]. The participating nursing students agreed to the interview process, and no one withdrew during the study.

The total duration of virtual simulation experiments and the average score of the experiments are exported from the background of the teachers’ account. The average score of the experiment can reflect the mastery of the knowledge in the experiment. Demographic information about the respondents is shown in Table 1.

**Table 1:** Demographic data describing the respondents (n = 14)

Number	Gender		Grade	Whether the internship/ probation unit is a provincial tertiary hospital	The total duration of virtual simulation experiments(min)	Average score of experiments (out of 100 points)	
A		woman	junior	Yes	468	89	
B		man	senior	No	175	73	
C		woman	senior	Yes	223	79	
D		woman	junior	Yes	499	92	
E		woman	junior	Yes	400	87	
F		woman	junior	Yes	271	80	
G		man	junior	Yes	254	84	
H		woman	senior	Yes	197	79	
I		man	sophomore	Yes	166	77	
J		woman	senior	Yes	212	83	
K		woman	junior	No	311	88	
L		man	sophomore	Yes	129	75	
M		woman	junior	Yes	245	79	
N		woman	junior	Yes	265	77	

### Interview outline

An interview outline was developed by considering previous literature reviews [20, 21] and expert consultation. The aim of this study is: (1) How do you feel about the learning experience within a virtual simulation experimental class? (2) Do you have any classroom experiences that impressed you? (3) What did you gain from the virtual simulation experimental class? (4) Compared with traditional offline courses, what advantages and disadvantages do you think virtual simulation classrooms have? (5) What are your opinions and suggestions for the virtual simulation classroom?

### Data collection methods

The interview location was chosen to be a relatively quiet location, without visual or audio interference. Before the formal interview, the investigator introduced the purpose, content and significance of this research to the interviewee, outlined the strict confidentiality principles of the responses, replaced the name with the letters A-N, obtained a signed informed consent form, and recorded the interviewing process.

The interview scene was recorded in full, paying attention to the expressions and body movements of the interviewee. Each interview was approximately 30 min in duration. All responses were recorded and collected until no new topics appeared.

### Data analysis methods

Using descriptive phenomenological research methods, a female researcher trained in qualitative interview techniques conducted in-depth interviews with nursing undergraduates in a face-to-face semi-structured interview method to understand their learning experience in virtual simulation experimental class. Data collection and analyses were undertaken simultaneously, and the recordings were converted into text within 24 h of the interview.

The data were analysed, sorted and summarised using a Colaizzi seven-step analysis method [22]: The interview recording was replayed many times enabling complete familiarity with the interview content, examining meaningful statements and determining significant results. The same topics were clustered into a class, coded and described in detail. The resulting information was returned to the respondent for verification, forming a final topic.

### Quality control methods

The interviewer has received systematic training in qualitative research, has proficient interview skills, and was supervised by an associate professor proficient in qualitative research methods. Researchers promptly reflected on each interview, reduced their influence on the results, and adjusted and supplemented the outline. After the interview, the research results would be confirmed and verified with the interviewee to ensure the authenticity and completeness of the data.

## Results

Among the respondents who participated in the study, ten were female, and four were male. The virtual simulation experiments considered during the interviews included an invasive ventilation experiment, a drowning experiment, a pre-hospital first aid experiment, a meridian acupoint experiment of traditional Chinese medicine, an infantile diarrhea experiment and a rehabilitation posture placement experiment. The above experiments were sorted according to the learning order of nursing students.

A case was imported for each type of experiment. The scenes of all cases had realistic animations and sounds, and they interacted with nursing students through dialogue. Nursing students could choose different scenes according to the prompts and answer questions according to the various scenes (for example, multiple-choice, fill-in, picture, and sorting questions). After all the scenes were completed, the total scores of the experiments were calculated and analysed.

Considering the theme and qualitative data analysis, two themes and six sub-themes of positive and negative experiences became apparent.

### Theme 1: positive experiences

#### The scene is real and diverse, with deep memory points

After the virtual simulation experiment had been completed, nursing students thought the diversity of experimental scenes brings them the most profound feelings, which increased their interest in learning while giving them an immersive experience and providing rigorous and standardised experimental steps and sequences.

Respondent A: “It should be drowning. There are many different situations, from discovering someone drowning to the final rescue in a hospital. Such as whether the rescuer can swim, the method of saving people, and the posture of the drowning person cramping in the water. This scene is very realistic. It feels like you are really in this situation and know how to do it (nods).“

Some nursing students noted the authenticity of patient behaviors and scene changes. The reality of the animation and sound immersed the nursing students in the experiment.

Respondent J: “There was a drowning experiment. The scene was too real, and there was a person in the water who was grabbing and shouting for help. We were the rescuers on the shore, following the process to guide us to make choices as if we had really experienced a rescue. After having mastered this skill, we could do it in real life (laughs).“

#### Virtual simulation experiments can break the time, space and location limitations

Nursing students said the virtual simulation experiment could experience the complete nursing operation by clicking the mouse, emulating a forward-looking experimental platform. Compared with traditional offline courses, virtual simulation experiments are no longer limited by time, space and location to a large extent.

Respondent I: “Going to the hospital for an internship in my next study plan, I will use the rest time to complete virtual experiments during the pre-service training. Following the instructor to practice can save time and be more meaningful.“

Notably, during any free time, the only two conditions of network and computer need to be negotiated, allowing unlimited opportunities to complete the experiment and assist in mastering the knowledge for nursing students.

Respondent C: “Because of the management of the experimental class, the offline experimental class does not allow us to practice additionally except for the class time. This platform still brings us much convenience.“

#### The display of knowledge is more logical, visualised and stereoscopic

Most nursing students emphasised that the knowledge in textbooks was too rigid, especially some professional nouns and complex knowledge points, and forced memory work did not enable them to understand and master the knowledge.

Respondent B: “For example, the acupuncture point positioning in Chinese medicine is directly presented on the model of the human body, and the things in the book are displayed in three dimensions, which is quite helpful.“

The virtual simulation experiment displays the knowledge in three dimensions, develops the nursing students’ systematic and logical thinking, and helps them integrate and fully absorb the knowledge.

Respondent K: “The 3D effect of virtual simulation experiments is advantageous. The sequence and logical level of the entire process of the experiment are clearer, which will be more three-dimensional than some textbooks on the class PPT.“

#### This is a popular trend

Virtual simulation experiments have been used as an auxiliary means of experimental teaching and are widely used in various disciplines. The disciplines and allied health professionals introducing virtual simulation experiments are gradually increasing. The content and form of virtual simulation experiments are gradually enriching with the characteristics of various disciplines. The number and popularity have also increased rapidly, reducing the loss of time and experimental material costs to a certain extent.

Nursing students pointed out that their progress and the completion of many courses are inseparable from virtual simulation experiment teaching and the familiarity with the virtual simulation experiment also increased with increasing numbers of training units.

Respondent C: “Due to the pandemic, many students cannot attend classes, but virtual simulation platforms provide alternative learning opportunities.

Respondent I: “I think virtual simulation experiments will be a trend in the information age. Its appearance certainly is for a reason, not only to shorten the cost of time but also to reduce consumable materials, which is very meaningful.“

### Theme 2: negative experiences

#### Learning efficiency varies by grade and subject

With increasing grade levels and virtual simulation courses, nursing students’ intellectual curiosity and freshness show a downward trend. The degree of difficulty in some virtual experiments is very high, and the process is too long. Learning efficiency is correlated with whether students take it seriously and whether the practice time is sufficient. Senior nursing students have a heavy academic burden and spend more time and energy completing the experimental modules, so they may be distracted and experience poor learning outcomes.

All respondents said the invasive ventilation experiment was time-consuming in the first virtual simulation experiment, the longest and generally taking more than one hour. The other experiments took about 40 min. In the follow-up practice, the time spent will gradually shorten by 5–10 min as the students complete more experiments.

However, the score should not evaluate the mastery of knowledge in the experiment. Some studies suggest that it is challenging to obtain psychological skills by completing virtual simulation experiments. Rather than providing patient care, the experiment focuses more on obtaining higher scores [23].

Respondent F: “Some experiments are too long to skip steps, and I will do it many times to get a satisfying score, and sometimes I am quite tired of it.“

In their narration, some nursing students also mentioned that if they are familiar with the knowledge points and processes before the experiment, it is conducive to the subsequent successful completion of the operation process. They believe a complete and feel-good experimental learning experience is inseparable from pre-class knowledge preview and familiarity.

Respondent G: “When encountering a long and uninteresting experiment, especially if it involves knowledge points you are unfamiliar with, you just want to end it quickly. If you do not have a good knowledge foundation before the class, you cannot learn much during the module.“

#### Inability to receive feedback in time for learning reflections

The virtual simulation experiment has many steps and tight logic. When the nursing student is inexperienced and wrong choices are made, there is no detailed explanation at the end of the experiment, resulting in the phenomenon that the knowledge learned cannot be summarised.

Respondent E: “When I make a mistake, I hope for an explanation instead of just telling me the choice is wrong.“

Reflective learning is an indispensable part of the experiment, which can help nursing students review the whole process and think deeply about the scenario and details. However, nursing students emphasised the lack of this module in the experiment, so the design of the reflective component of the virtual simulation experiment requires improvement.

Respondent M: “The invasive ventilation experiment is very complex and error-prone, but I hope that in the process of repeating it, I can make progress over and over again. This knowledge point can be more complete for me, and no longer fragmented.“

## Discussion

### Analysis of virtual simulation class experimental teaching and learning experience by undergraduate nursing students

#### Analysis of positive experiences

This study investigates the hypothesis that the virtual simulation experimental class can provide a variety of experimental simulation scenarios for nursing students and stimulate the nursing students’ interest in learning and operation desire by adding human-computer dialogue, music and other components. This improves participation and interactivity while facilitating the after-class review of nursing students, countering any time and space limitations.

When students cannot go to clinical practice or internship, virtual simulation class experimental teaching can be used as an auxiliary or supplementary education means to create an authentic and risk-free environment to impart knowledge. The knowledge points from books are displayed in virtual simulation experiments, broadening the space of thinking and imagination and changing the learning mode from teacher-led to students’ independent learning.

As Jeffries Simulation Theory highlights, teachers play the role of facilitators in the learning process, simulating students’ autonomy and enthusiasm to the greatest extent, strengthening their mastery of knowledge, and improving the success rate of traditional practical operations [24–26].

Other studies believe that virtual simulation is becoming increasingly popular in nursing education, which can reduce time and cost to a certain extent, effectively improve nursing students’ learning efficiency and quality, and provide a more culturally diverse and inclusive learning experience. It positively improves nursing students’ knowledge, skills, clinical reasoning and judgment to provide patients with high-quality care, which is the same as the views expressed by some respondents [27–29].

With the issuance of various documents [30], virtual simulation class experimental teaching, as an essential part of online learning carried out by universities since the epidemic in 2020, has played an indelible role in the innovative development of future educational methods [31].

#### Analysis of negative experiences

Different disciplines have different emphases, and knowledge points can be too fragmented, showing the differences in virtual simulation experiments of various disciplines. Moreover, nursing students cannot get teacher feedback following the initial pass through the virtual module and summarising learning points. It is easy to fall into a cycle of mistakes- where inaccuracies and mistakes are ignored and remade.

Students facing heavier learning tasks can resist the load and reduce engagement, resulting in inefficient learning, which is consistent with the findings of Eltaybani and other scholars [32]. Furthermore, the virtual simulation experiment has the uncontrollability of online learning. It is easy to forget the original learning task after being distracted in the free network environment [33].

With the low execution of some students, their insufficient understanding of the teaching method, and lack of attention to the experimental results, a marked reduction in learning outcomes and resource utilisation can occur.

This study shows that the longer the experiment time, the more likely the nursing students will develop anxiety and increasing irritability. However, Sim and other scholars believe that when the virtual simulation experiment time exceeds 30 min and the clinical scene frequently changes, it is more conducive for learners to improve their clinical reasoning skills [34]. The reason for such differing views may be that previous studies associate importance with providing feedback after the experimental scene, allowing nursing students to reflect on the learning outcomes.

In summary, reducing the interference of the network environment, improving students’ execution and learning efficiency, formulating more practical learning tasks and more perfect reflective learning components after cases to promote the development of nursing students’ clinical skills and other issues urgently need to be addressed in future research and virtual reality teaching modules.

### Improve the construction of virtual simulation class experimental teaching projects

#### Strengthening the information technology support is beneficial to the environment construction in virtual simulation class experimental teaching

According to Yi Sun, Bathini P and other scholars [35, 36], online learning is not the mainstream form of education in China. However, it is undeniable that education informatisation has entered the 2.0 era, with an emphasis on integration and innovation. With the development of various electronic technologies, information technology has gradually penetrated various fields, thus breaking the limitations of traditional experimental teaching, and virtual simulation experiments have emerged [37].

The results of one study indicate that compared with offline experimental courses, virtual simulation experiments have the convenience of computers and are more convenient for obtaining information [38]. Therefore, nursing students can obtain more effective and attractive learning resources to better adapt to unknown and complex clinical environments.

However, virtual simulation technology can make up for the shortcomings of some experiments that cannot be carried out in the laboratory environment and has the characteristics of flexibility, intuitiveness, networking and intelligent management [39, 40]. Such virtual simulations provide an opportunity to simulate clinical practice, improving nursing students’ sensitivity in the face of clinical problems and the correctness of problem decision-making.

Some nursing students pointed out that the fluency and simulation degree of virtual simulation experiments greatly depend on network program hardware problems, and it is easy to affect the learning outcomes due to network problems causing screen lag. This finding is consistent with previous studies by Foronda and others [41], who reported that network technology is prone to failures and problems, affecting teaching quality when conducting virtual simulation experiments. At the same time, the learning experience of nursing students is disrupted, which may lead to depression and anxiety. Hence, teachers need to pay more attention to the emotional expression of nursing students.

Therefore, the optimal hardware is fundamental for achieving ideal learning outcomes and experiences. With information technology’s continuous development and improvement, solving network problems is just around the corner and needs to be verified in future studies.

#### Following national education policy is conducive to the popularisation of virtual simulation class experimental teaching

The development of virtual simulation experiments should be synchronised with national policies. In China, for example, since 1999, online education piloted by more than 60 colleges and universities has become an integral part of China’s higher education. The policies issued by the Ministry of Education between 2012 and 2018 aim to promote the construction of virtual simulation class experimental teaching. The teaching focus has changed from “teaching” to “learning”, showing its unique value [42].

The vigorous promotion of virtual simulation experiments conforms to the development trend of higher education. It is the latest measure of experimental teaching informatisation in colleges and universities and will play an essential role in improving the quality of higher education in our country.

In addition to the guidance and supervision of the school, the unified curriculum quality and teaching effect as the goal and direction can also promote cooperation and development between schools and enterprises, introduce and train high-level teachers, and form a virtuous circle of closed-loop evaluation of teaching quality [33, 43]. Such broad and mainstream implementation will support students in the classroom to accept virtual simulation experiments and then promote their publicity and popularisation.

#### The development of virtual simulation experiments improves teaching quality and puts forward higher teacher requirements

Coding analysis of the interview data reveals that introducing virtual simulation experiments promotes the development of nursing education, brings new challenges, and puts forward higher teacher requirements. Virtual simulation experiments attach importance to various clinical scenarios that nursing students may face in future clinical practice or probation, which helps reduce adverse clinical events [18].

In the teaching process of simulation experiments, teachers play the role of learning promoters. Their task is to provide efficacious experimental design and equipment to support learners [4]. This study shows that teachers need timely guidance, error correction and summary, without missing knowledge and critical points. Only by promoting the deep learning of nursing students and meeting the standards of effective teaching can nursing students have the best learning experience and educational results [44]. Therefore, teachers are an indispensable factor in the success of the simulated class.

In the information age, it is a new challenge and teaching pressure for teachers to use the new network teaching platform to design experimental scenes and plan reasonable virtual simulation experiment hours and forms. Education policymakers should optimise teaching management measures and reasonably arrange teachers’ tasks. Teachers should adjust their own positioning in time and constantly consider and explore how to better exercise nursing students’ dialectical thinking and practical operation abilities.

#### Combined with the characteristics of disciplines, bringing its superiority into full play is conducive to the diversified development of virtual simulation class experimental teaching

Some nursing students said that undertaking a nursing major degree is often confusing initially. Nurses are a profession that needs to respond to various accidents and challenges at all times. However, to a certain extent, virtual simulation experiments can help increase their confidence in facing future emergencies, improve their understanding of the nursing specialty, enhance their professional identity, and cultivate nurses’ professional quality [25, 45].

Moreover, the experiment is instructive, simulating the dialogue with patients and patient’s families from a first-person perspective, gaining the sense of identity of the student group, making them pay more attention to humanistic care, giving full play to their personality characteristics, and improving their humanistic literacy while improving academic literacy [46].

The virtual simulation experiment takes “the real is not virtual, the combination of the virtual and the real” as the guiding principle, breaking the shackles of traditional experimental teaching. Goldsworthy and other scholars believe that clinical practice cannot guarantee the opportunity for nursing students to contact patients with deteriorating conditions. However, virtual simulation helps nursing students learn to deal with emergencies, a novel teaching method [47].

In summary, virtual simulation experiments have promoted the diversification of teaching modes, changed traditional teaching strategies and educational methods, greatly enhanced the subjective initiative of nursing students, and cultivated more and more innovative talents for the country [48–50].

## Study limitations

The limitations of this study are predominantly related to the number of nursing students who participated in the interview and the qualitative research methods. This study aims to explore the active learning experience of nursing students in a virtual simulation experiment. Future research can include more participants or conduct randomised controlled trials to investigate the advantages of virtual simulation experiments in improving nursing students’ knowledge and practical skills. Despite these limitations, qualitative responses were substantial and represented essential elements of the Jeffries Simulation Theory.

## Conclusion

Virtual simulation technology has gradually appeared in the experimental teaching modes of national and international universities. This innovative teaching mode not only improves the efficiency of teaching management but also provides convenient conditions for implementing experimental classes, an inevitable trend in the information age and a powerful supplement to education.

There is no doubt that the perfect presentation of virtual simulation technology in teaching quickly satisfies students’ thirst for knowledge, and the teaching outcomes increase sharply, allowing for the exploration of combining information technology and medical fields to unleash the full imagination. Today, cutting-edge technologies such as virtual simulation technology and the metaverse yield a clear vision of future teaching models undergoing earth-shaking changes.

Virtual simulation experiment has not yet been widely used in nursing courses. It is necessary to solve how to reduce the interference of the network environment, how teachers can adjust their own positioning to formulate more reasonable learning tasks to improve the learning enthusiasm of nursing students, and how the knowledge in the experiment can be more integrated with the knowledge in the book.

In future research, the development of nursing courses and virtual simulation experiments should be carried out simultaneously to avoid the impact of experimental lag as much as possible, enabling the improved evolution of virtual simulation class experiment teaching and learning projects.

## Data Availability

The datasets generated and/or analysed during the current study are not publicly available but are available from the corresponding author upon reasonable request.
